# Challenges and opportunities for improving access to adolescent and youth sexual and reproductive health services and information in the coastal counties of Kenya: a qualitative study

**DOI:** 10.1186/s12889-024-17999-9

**Published:** 2024-02-15

**Authors:** Evaline Chepchirchir Langat, Abdu Mohiddin, Flaura Kidere, Anisa Omar, Job Akuno, Violet Naanyu, Marleen Temmerman

**Affiliations:** 1 Center of Excellence in Women and Child Health, Aga Khan University, East Africa, Kenya; 2 Department of Gender, Culture, Social Services and Sports, County Government of Kilifi, Kilifi, Kenya; 3 Pwani University, Kilifi, Kenya; 4 Elizabeth Glaser Paediatric AIDS Foundation, Washington, DC, USA; 5 School of Arts and Social Sciences, Moi University, Eldoret, Kenya

**Keywords:** Adolescents, Youth, Sexual and reproductive health, Focus group discussions, Kenya

## Abstract

**Background:**

Globally, adolescents and youth experience high unmet need for sexual and reproductive health (SRH) information and services. In Kenya, evidence shows that more than half of teenage pregnancies are unintended and that half of all new HIV infections occur in people ages 15-24-year-olds, with the majority of those being female. The coastal counties in Kenya record a relatively high adolescent pregnancy rate and higher rates of unmet need for contraception for all women of reproductive age compared to the national average. This study focused on gaining a deeper understanding of the existing challenges to and opportunities for accessing SRH information and services among adolescents and youth (AY) at the Kenyan coast.

**Methods:**

Using qualitative methods, this study conducted thirty-six focus group discussions with adolescents, youth, and community health volunteers across all the six coastal counties in Kenya. The sample included adolescents aged 10–14 years in school (male and female), adolescents aged 15–19 years not in education (male and female), youths aged 20–24 years (mix of both male and female), and community health volunteers who were conveniently sampled. Thematic analysis was used to examine the data and report the study results.

**Results:**

The barriers to accessing AYSRH identified in the study are individual factors (feelings of shame, lack of information, and fear of being judged) parental factors, healthcare worker and health institution factors, teacher/educators factors, and broader contextual factors such as culture, religion, poverty, and illiteracy. Factors that facilitate access to AYSRH information and services included, supportive parenting and culture, AYSRH sessions in schools, peer support, supportive health institutions, gender inclusivity, and digital technology.

**Conclusions:**

AYSRH information and services at the Kenyan coast is strongly influenced by a range of individual, social, cultural, and economic factors. Improving access to AYSHR necessitates meaningful AY engagement, provision of youth-friendly services, use of digital technology as alternative pathways for sharing SRH information, strengthening parent-AY relationships, embracing peer-to-peer support, and the adoption of gender-inclusive approaches in AYSRH programming.

## Introduction

Adolescents and youth represent 27% of the world’s eight billion population [1] and 60% of the population in Africa [2]. The World Health Organization (WHO) defines adolescent sexual and reproductive health as the physical and emotional well-being of adolescents and includes their ability to remain free from unwanted pregnancy, unsafe abortion, sexually transmitted infections (STIs) including HIV/AIDS, and all forms of sexual violence and coercion [3].

Globally, and in Kenya adolescents (10–19 years) and youth (20–24 years) have a high unmet need for sexual and reproductive health (SRH) services and information [3, 4]. Evidence show that adolescents and youth are disproportionately vulnerable to increased risk of HIV infection and unintended pregnancy due to their lack of social and economic protection as well as the developmental, psychological, social, and structural changes that converge during this period [5].

In Kenya, 59.2% of its population are below the age of 24 years with 23% being between the ages of 10–19 years [6]. Like other low-and middle-income countries (LMICs), adolescents and youth in Kenya face several challenges to their lives and general well-being. These challenges include vulnerability to early and unintended pregnancy, unsafe abortion, female genital mutilation (FGM), child marriages, gender-based violence (GBV), malnutrition, and reproductive tract infections including sexually transmitted infections (STIs) [7].

## Adolescents sexual and reproductive health performance in Kenya

Sexual debut starts early in Kenya, with 21.5% of young people reporting engaging in sexual intercourse before the age of 15 years, with the rate being higher (30.3%) among adolescent boys and young men compared to adolescent girls and young women (12.6%) [8]. Early pregnancies among those aged 15–18 years are equally reported, with one in ten of the adolescent girls in this age group giving birth to one or more children [8]. The unmet need for contraception among adolescents and young women is higher for those who are unmarried, 34.5% and 21.1%, compared to married, 21.6% and 16.9%, respectively [9]. HIV incidence among adolescents and youth in Kenya accounts for 50% of all new infections, although their prevalence is lower (1.4%) than that in the adult population (4.9%). HIV testing coverage performance among adolescents and youth is reassuring for both adolescent girls and young women (67%), as well as adolescent boys and young men (50.5%) [9].

This study aims to gain a deeper understanding of the challenges to and opportunities for improving access to SRH information and services among adolescents and youth (AY) in the coastal parts of Kenya. This paper presents part of ongoing work at the Jumuiya ya Kaunti za Pwani (JKP) WHO adolescent and youth sexual and reproductive health and rights (AYSRHR) technical assistance (TA) programme. The findings presented here will be useful in the next phase of TA, which includes co-developing a regional AYSRH strategy and county specific implementation plan.

## Method

### Study sites

The study focused on all six coastal counties (Mombasa, Tana River, Taita Taveta, Lamu, Kwale, and Kilifi), all of which fall under JKP, a regional economic development bloc [10]. Despite being in the coastal region, these counties have distinct contexts, which are detailed below.

**Mombasa County** is a largely urban cosmopolitan county, with an estimated population of 1,208,333 [5]. The AY population constitutes 29% of the total population in Mombasa, of which 11,5424 (9.6%) are those between the ages of 10–14 years, 10,0733 (8.3%) are aged 15–19 years, and 139,733 (11.6%) are between the ages of 20–24 years [5]. The main ethnic communities include Mijikenda, Swahili, and Kenyan Arabs. Mijikenda is the largest community in Mombasa County, accounting for almost 35% of the county’s total population. Immigrant communities include Asians and Kamba, with the Kamba community forming the second largest ethnic community in the county, accounting for almost 30% of the total population of the county.

**Kwale County** has an estimated population of 866,802 [5]. The AY population constitutes 34% of the total population in Kwale of which 125, 289 (14.5%) are between the ages of 10–14 years, 97,104 (11.2%) are between 15 and 19 years and 71, 795 (8.3%) are between the ages of 20–24 years [5]. Kwale is mainly an inland county, but it also has a coastline south of Mombasa. The main ethnic communities in the county include the Digo and Duruma clans of the larger Mijikenda tribe and also a significant presence of the Kamba tribe. Digos are the majority in Msambweni, Lunga, and Matuga, while Durumas are dominant in Kinango. Most Kambas are found in Kinango and Lunga, with a significant population in Msambweni. Other significant immigrant communities included the Luo, Luhya, and Somali communities.

**Kilifi County** is largely a rural area on the Kenyan Coast, 60 km north of Mombasa County, with a population of 1,453,787, according to the 2019 national census [5]. The AY population constitutes 35% of the total population in Kilifi, of which 206,722 (14.2%) are between the ages of 10–14 years, 172,350 (11.9%) are between 15 and 19 years, and 135,391 (9.3%) are between the ages of 20–24 years [5]. The residents of Kilifi County are mainly Mijikenda, a Bantu group of nine tribes, with Giriama (45%), Chonyi (33%), and Kauma (11%). The main economic activities are subsistence farming and fishing, and the average monthly income per person is roughly 700 Kenyan Shillings (US$8). Approximately 55% of the population in Kilifi County is regarded as having low socioeconomic status, with 62% of the population having low literacy levels.

**Lamu County** is located on the Northern Coast of Kenya and borders Tana River County to the southwest, Garissa County to the north, Republic of Somalia to the northeast, and the Indian Ocean to the South. It has an estimated population of 143,916 people, according to the 2019 National Census [5]. The AY population constitutes 32% of the total population in Lamu, of which 18,473 (12.8%) are between the ages of 10–14 years, 15,152 (10.5%) are between 15 and 19 years, and 13,170 (9.2%) are between the ages of 20–24 years [5]. The main economic activities in the county include crop production, livestock production, fisheries, tourism, and mining, most notably, quarrying. Among the challenges facing Lamu is population growth owing to migration into Lamu from other parts of the country, fueled partly by the anticipated opportunities accruing from the Lamu Port South Sudan-Ethiopia Transport (LAPSSET) Corridor.

**Tana River County** is named after the Tana River. It has a population of 315,943 according to the 2019 national census [5]. The AY population constitutes 34% of the total population in the Tana River, of which 46,690 (14.8%) are between the ages of 10–14 years, 32,935 (10.4%) are between 15 and 19 years, and 27,176 (8.6%) are between the ages of 20–24 years [5]. The major ethnic groups are the Somalis, Pokomo (many of whom are farmers), the Orma, and the Wardey. The county is generally dry, and prone to drought. Conflicts have occurred between farmers and other people with access to water. Flooding is a common problem caused by heavy rainfall in the upstream areas of the Tana River. The Tana River County presents an interesting case of the nexus between conflict and food security.

**Taita–Taveta County** lies approximately 140 km northwest of Mombasa and 380 km southeast of Nairobi County. The population of the county is estimated at 340,671 according to be 2019 national census [5]. The county covers an area of 17,083.9 km2, of which 62% (11,100 km2) is within the Tsavo East and Tsavo West National Parks. The remaining 5,876 km2 is occupied by small-scale farms, ranches, sisal estates, water bodies (such as Lakes Chala and Jipe in Taveta and Mzima springs), and hilltop forests.

The JKP regional bloc has an estimated total population of 4,112,585 [5], and records varying differences in AYSRH outcomes. The Tana River and Taita Taveta counties have relatively high adolescent pregnancy rates of 17.6%and 18.4%, respectively, compared to the national average of 15%, whereas Kilifi, Mombasa, Lamu, and Kwale counties have better performance on the same indicator at 12.5%, 10.8%, 13.7%, and 14.8%, respectively [9]. On the other hand, Mombasa and Taita Taveta reported high HIV prevalence rates of 5.9% and 5.2%, respectively, in the entire population compared to the national prevalence of 4.9% [8]. Regarding the unmet need for contraception for all women of reproductive age, the Tana River and Kwale record the highest rates at 34% and 24%, respectively, compared to the regional rate of 21% and the national average of 14% [9].

### Sampling and recruitment strategy

A qualitative design was used to address the objectives of this study. The study population comprised school-going adolescents aged 10–14 years (male and female), adolescents not in education aged 15–19 years (male and female), youths aged 20–24 years (male and female), and a mix of both male and female community health volunteers (CHVs). Convenience sampling was used to select participants within each group.

The study participants were selected after a letter was sent to the heads of both county and national health, education, and gender sectors and the health sector within the six coastal counties from the JKP secretariat detailing the purpose of the study. Members of the research team from AKU and JKP visited the participating counties and were linked to the focal person who then mobilized the targeted participants from the villages and schools according to the study inclusion criteria (adolescent males and females aged 10–19 years, youths aged 20–24, residents of the six [6] counties for more than one year, willingness to take part in the study by providing informed consent or assent, and CHVs working within the six counties) and exclusion criteria (children below 10 years and non-residents of the six [6] counties for more than one year).

Those who agreed to participate in the study were invited to meet with the research team in the designated areas in each county (either a health facility or a school that was considered a safe space for the participants), where they provided consent to be involved in the study after they had been duly informed about the study using the approved information and consent forms. For minors (under the age of 18 years), the legal guardians were mobilized to accompany the participant during the focus group discussion and were provided with information about the study. For minors who indicated interest in participating, parental consent was signed, and the minors’ assent was obtained. Once the consenting process was completed, the focus group discussion was undertaken in a separate room where parents were not part of the focus group discussion and allowing the participants to speak freely.

### Ethical considerations

Ethical approval was obtained from the Aga Khan University Nairobi Institutional Scientific and Ethics Review Committee 2022/ISERC-30(v1), Pwani University ERC/EXT/003/2021, and National Commission for Science Innovation and Technology NACOSTI/P/22/15,214. Administrative approval from all six coastal counties was obtained before commencing data collection.

All the respondents provided informed consent prior to participation. Those who agreed to participate signed two copies of the consent document and were given a copy of the study information and contact details to take home. All notes, transcripts, and audio recordings were stored in password-protected files and were only accessed by the study team. All data used in the reports were de-identified to ensure the anonymity of the participants.

### Study tools and data collection

The interview guides used for data collection were based on the available literature on the factors influencing access to AYSRH information and services [7, 11–15]. The broad domains captured in the data collection tools were perception and experience of facility readiness in terms of the overall capacity to provide AYSRH services (equipment supplies and medicines); availability relating to the physical presence of facilities, resources, and services for AYSRH services; accessibility in terms of geographic and financial aspects; and individual and contextual factors.

Data were collected by a research team comprising of two supervisors and two trained research assistants. Research Assistants (RAs) were competitively recruited by the study coordinator on the basis of expertise, education, and knowledge and held a minimum diploma in health or social sciences. The study coordinator trained the field team and took them through the inclusion criteria, consent processes, data collection methods, study tools, and field logs to be used. All data collection sessions were conducted face-to-face and were captured using audio recorders and short-hand notes. Fieldwork was implemented between 11th July to and August 4, 2022. Upon arrival, research assistants sought informed consent and engaged respondents for approximately 1 h in open-ended dialogues on the study content. Each FGD was carried out separately (male FGD separate and female FGD separate) by a qualified facilitator and a note-taker (male and female).

### Data management and analysis

All interviews were conducted in Kiswahili, and the audio recordings were transcribed verbatim and translated into English. Two data analysts used NVivo Version 12 software to code and analyze the transcripts [16]. Thematic analysis was performed following the six steps identified by Braun and Clarke [17]. The first step of the analysis included reading and rereading all interview transcripts and notes to aid in familiarization with the entire data corpus while drafting notes about the initial impressions. The second step involved the generation of initial codes, enabling the data to be organized in a meaningful and systematic manner. The third step entailed organizing the different codes into themes of challenges, barriers, and opportunities for AYSRH. Several codes related to each theme have been merged.

The fourth step entailed reviewing, modifying, and developing the themes identified in Step 3, taking note of whether the themes made sense, whether the data supported the themes, and considerations for condensing or expanding the themes. In the fifth and sixth steps, the themes were examined in relation to the objectives of the study.

To establish reliability of the study findings, this study used the multiple coders approach where two coders independently reviewed the codes of the first interviews and met to discuss any agreements and disagreements and where coding discrepancies occurred, the coders examined the transcripts together until they reached agreement and refined the codebook. Saturation in this study was also established at the analysis stage. where no new meaning was being derived for the codes was the agreement of saturation arrived at. The data analysis process was guided by the anthropologist in the team who doubled checked the codes and the transcripts at various points in the data analysis process.

## Results

### Participants characteristics

A total of 36 FGDs engaging 358 participants were conducted. As shown in Table 1, there were 12 FGDs with adolescents aged 10–14 years in school (58 males and 60 females), 12 FGDs with adolescents aged 15–19 years not educated (60 males and 60 females), 6 FGDs with youths aged 20–24 years (10 males and 50 females), and 6 FGDs with community health volunteers (9 males and 51 females). There were two refusals for adolescent male 10–14 years of FGD, where the minors refused to participate despite parental consent. The reason cited for refusal was discomfort in being part of the group discussions. We did not replace these refusals because the number of participants was above the minimum of six members to hold a robust group discussion.

**Table 1:** Participants characteristics and sites

Targeted sessions						
	Mombasa	Kilifi	Kwale	Tana River	Taita Taveta	Lamu
12 FGDs with adolescents aged 10–14 years (male and female)	2 FGDS	2 FGDs	2 FGDS	2 FGDs	2 FGDS	2 FGDs
12 FGDs with adolescents aged 15–19 years not in education (male and female)	2 FGDS	2 FGDs	2 FGDS	2 FGDs	2 FGDS	2 FGDs
6 FGDs with youths aged 20–24 years (mix of both male and female)	1 FGD	1 FGD	1 FGD	1 FGD	1 FGD	1 FGD
6 FGDs with community health volunteers	1 FGD	1 FGD	1 FGD	1 FGD	1 FGD	1 FGD

The results of the focus group interviews are described in the following paragraphs and further illustrated by quotes of the participants in Tables 2 and 3 (Q references in the text refer to quotes of specific themes in Tables 2 and 3).

**Table 2:** Illustrative quotes related to barriers to accessing SRH information and services among the AY

Theme	Quote	
Individual factors	Q1	Sometimes people feel shame approaching other people asking about these things. There is a kind of shyness in people that prevents them from asking. (Male FGD, 15–19 Years, Kilifi County)
	Q2	There are some situations - not that one is prevented - but one feels shame to go to a place and ask for such information. They see it as shame, they have no confidence to sit with other people [and ask them] to give them such information. (Youth FGD, 20–24, Lamu County)
Parental factors	Q3	If I ask my mother now, ‘Can I do this? Or how can this be done?’ She will deny it. She will tell me she cannot give me such information until I get married. They also ask you, ‘Why do you want to know about that? What do you want to do?’ They advise you ‘Get married first, get a child, and we will tell you about that.’ If you want pads they will buy for you, but they will not give you information. (Adolescent girls FGD, 15–19 Years, Lamu County)
	Q4	Sometimes a parent gives information to their children late when they are already in problems. (Boys FGD, 10–14 Years - Taita Taveta County)
	Q5	I think only a few parents can give SRH information to their children because they think if they give them information about FP, they will go and test on that, a parent telling their children about the use of condoms is like they are encouraging them to use condoms. (Youths FGD, 20–24 Years, Mombasa County)
	Q6	Parents think if they tell you how to prevent pregnancy, you will engage into sex and then prevent yourself. (Boys FGD, 10–14 Years, Lamu County)
	Q7	They think if they give you such information it is like they enable you to go and engage in sex without fear of getting pregnant. (Adolescent girls FGD, 15–19 Years, Kwale County)
	Q8	Parents feel that if they give such information to their children is like encouraging them to engage into sex. For me personally I cannot tell my child that if you do this you will not get this, it is you - you are showing the child how to do it. (CHVs FGD, Mombasa County)
	Q9	Mothers feel shy and ashamed discussing sexual information with their male children. (Girls FGD, 15–19 Year. Mombasa County)
	Q10	Parents also see giving such information to their children as shameful. (CHVs FGD, Lamu County)
	Q11	Some young people don’t have people to guide them, they don’t have parents and they don’t go to school. (Boys FGD, 10–14 Years, Kwale County)
	Q12	Parents are busy, they go to work, every morning you wake up they are not there, and they come back late. (Adolescent girls FGD, 15–19 years, Taita Taveta County)
	Q13	Parents go out early in the morning at 6:00 am and come back late in the evening at 9:00 pm when the children are asleep and does not get time to talk to them. (Youth FGD, 20–24 Years, Taita Taveta County)
	Q14	Some parents don’t have time with their children because they are busy and always think there will be another day to share the information and the child continues to grow getting destroyed behaviorally but [parents] will never sit with them to give [SRH] information (CHVs FGD, Kilifi County)
	Q15	Many people here are Muslims and mothers are not allowed to teach their sons such things. They may get such information from an uncle. (Adolescent girls FGD, 15–19 Years, Kwale County)
	Q16	Like girls are not allowed to go out to be given such information, their parents are harsh. You find that a girl gets a chance only when the father is not at home, so they never get information. They can only get out when their parents are away. Because of that, they don’t get the information which they are supposed to be given as girls, you find a girl is not comfortable and doesn’t know anything. (Adolescent girls FGD, 15–19 years, Kilifi County)
	Q17	Because culture does not allow [a mother] to give such information to her son (Adolescent girls FGD, 15–19 Years, Kilifi County)
	Q18	Sometimes the parent and son may not be in good times. The son always comes back home under the influence of drugs and refuses to listen to the parent. (Boys FGD, 15–19 years, Taita Taveta County)
	Q19	Parents assume that as youths we already have the information so there is no need for them to tell us anything about SRH. (Youth FGD, 20–24 Years, Taita Taveta County)
	Q20	Another barrier is illiteracy. In most families you find the children are more educated than the parents. In that case a parent assumes that the child has all the information they require. (CHVs FGD, Kilifi County)
Health care worker and health institution factors	Q21	Sometimes you may want to inquire about safe sex from a dispensary but you find that whoever is there is an old man and cannot tell you about this. (Male FGD, 15–19 Years, Kilifi County)
	Q22	It is not free because if you come to hospital, even just seeing the doctor to explain your problem is charged, if you go to lab, you also pay. (Adolescent girls FGD, 15–19 Years, Mariakani)
	Q23	If you go to hospital and you don’t have money you will not get treated, you go back home with your illness. (Adolescent girls FGD, 10–14 Years, Lamu County)
	Q24	Some hospitals don’t give services on Sundays, for example, I fell sick and went to hospital and they told me it was a Saturday, and they don’t give services. (Adolescent girls FGD, 10–14 Years, Lamu County)
	Q25	There are no services during weekends - The doors are open, but they tell you they can only see emergency cases. (Youth FGD, 20–24 Years, Kwale County)
	Q26	Services are given between 8:00 am and 4:00 pm, beyond that time [there are] no services. If you come, you are told they have closed. (Adolescent girls FGD, 15–19 Year Mombasa County)
	Q27	Hospitals also lack enough doctors… You may take a patient there at 9:00 am but the doctor will arrive at the hospital at 1:00 pm. (Youth FGD, 20–24 Years, Taita Taveta County)
	Q28	Some places in Lamu are far away from the hospital and transportation problem prevents people from coming here. (CHVs FGD, Lamu County)
	Q29	Not all people can easily reach them [health facilities]. Some people live far away and must use transport to get to hospital. (Adolescent girls FGD, 15–19 years, Taita County)
	Q30	Many of them live far away from hospitals and cannot easily reach there. (Boys FGD, 10–14 Years, Tana River)
Teacher/educator factors	Q31	It is this way, teachers give us information, but they say they cannot teach us some other things because they are not relevant at our age. (Adolescent girls FGD, 10–14 Years, Kwale County)
	Q32	They don’t give enough information, things like STIs, they know we are students and cannot dwell on that. But we come from different backgrounds. Maybe some have already been involved in those things, but they decide not to tell them, and they could be already infected with gonorrhea. (Adolescent girls FGD, 15–19 Years, Kwale County)
	Q33	I think they [teachers] are avoiding discussing some topics to avoid stigmatization from parents. When children are given such information, they also disclose to their parents who go to school to attack teachers. So a teacher cannot risk losing her job and that is why they avoid some topics. (Youths FGD, 20–24 Years, Mombasa County)
	Q34	I think they have the information, but they fear if they give them full information, they will tell other people that the teacher told me this and that. (CHVs FGD, Tana River County)
	Q35	We have inadequate time allocated for school’s SRH sessions (Male FGD, 15–19 Years, Mombasa County)
Contextual factors	Q36	…That is also the same even to our mothers, others cannot read, they don’t know anything, and this contributes a lot. (Adolescent FGD, 15–19 Years, Lamu County)
	Q37	Normally as youths, we can discuss such things, but in some places, like in the rural areas, you will find youths are illiterate so none of them can give information to the other. (Youth FGD, 20–24, Lamu County)
	Q38	Islamic religion does not allow discussion of such things. It’s not easy. It is not allowed because a girl who is not married is not allowed to have sex. (Adolescent girls FGD, 15–19 Years, Kwale County)
	Q39	The boys are given information by their fathers or grandfathers and the girls are given information by their mothers or grandmothers. (Adolescent Boys FGD, 15–19 Years, Kwale County)
	Q40	The poor are lonely most of the time because people look for friends who have similar social back grounds. (Adolescent girls FGD, 15–19 years, Taita County)
	Q41	Once you have a lot of responsibilities, you don’t get time for other things…. Take an example of a girl whose parents are poor and she is left at home to do all house work as parents go out to look for a living, how will she get time?…Already her mind is blocked, she cannot think further. (Youth FGD, 20–24 Years, Mombasa County)
	Q42	Children from poor families also go out to look for food for their families. Sometimes there could be a meeting in their areas that discusses SRH information, but because they have to go to work, they will miss that opportunity to attend. (Male Adolescent FGD, 15–19, Kilifi County)
	Q43	If a young person is asked to come here but stays far away and does not have fare, there is no money at home, that person will not be able to come and get full information. (Adolescent girls FGD, 15–19 Years. Mombasa County)
	Q44	The rich always have time with their children but the poor parents always go out to look for food. They go looking for work, but the rich are already employed and sometimes get free time to sit with their children and advise them. (Adolescent girls FGD, 15–19 Years, Kwale County)

**Table 3:** Illustrative quotes related to factors facilitating access of SRH information and services among AY

Theme	Quote	
Supporting AYSRH programs in schools	Q45	Apart from teachers giving SRH information, there are also organizations that visit students. I am even one of them. I have a group of other people, and we visit schools and request for permission to give students information (CHVs FGD, Tana River County)
	Q46	Guiding and counselling people are brought to schools to teach. (Adolescent girls FGD, 15–19 Years, Kwale County)
	Q47	A long time ago doctors from King Fahad hospital came and talked to us about drugs. They told us if a pregnant woman takes drugs, the baby will get affected. (Adolescent Boys, 10–14 Years, Lamu County)
Supportive Parenting	Q48	According to how people are brought up here, when you reach puberty, your parents will tell you that ‘You are now a grown-up person, so you need to do this and that.’ That is the parent telling you that if you do this you will get a certain problem. (Youth FGD, 20–24, Lamu County)
	Q49	I think it depends on the relationship between parents and the children. In some families, people discuss issues openly, and if one has a problem, it’s easier for them to inform their parents and they will recommend going to hospital. But for those who are restricted, they are isolated and have little interactions. If such youths get a small opportunity, they mess up. (Youths FGD, 20–24 Years, Mombasa County)
Peer support	Q50	For those who are friends, they share a lot of information and even their problems. (CHVs FGD, Kilifi County)
	Q51	It’s easy for them to share information among themselves. (CHVs FGD, Lamu County)
	Q52	It’s rare to find a parent giving SRH information to their children, so we learn from experience or from friends. (Youths FGD, 20–24 Years, Mombasa County)
	Q53	My elder brother told me if I use a condom, I will prevent pregnancy. (Adolescent boys FGD, 10–14 Years, Lamu County)
Supportive health system	Q54	Here [health facility], people are given information. Before start of services, people are taught about different things, like menstruation hygiene, about FP. They talk to the mabinti (young girls) if they are there, but they also talk to mothers so that they can convey the messages at home. (CHVs FGD, Kilifi County)
	Q55	Some doctors understand, if you tell them what you feel. They will ask you what you did and help. But some will be arrogant to you and ask you ‘Why did you do it?’’ (Adolescent girls FGD, 15–19 Year. Mombasa County)
	Q56	I think TVs give more information because information could be given by a professional health care worker on RH, but maybe the parents did not even step in a classroom and do not know many things. They will only tell you a few things which they may know and leave others which you will leave you ignorant about them. (Adolescent girls FGD, 15–19 Years, Kwale County)
	Q57	There were assumptions that girls went through more challenging SRH experiences in comparison to boys; consequently, they needed to be knowledgeable and protect themselves, for example, from unintended pregnancy (CHVs FGD, Lamu County).

### Barriers to access of SRH information and services among the AY

The factors that were identified as potential barriers to accessing SRH information and services were grouped into five broad themes: individual, parental, healthcare worker and health institution, teacher, and broader contextual factors. The individual factors were mainly mentioned by both male and female participants aged 15–19 years and youths ages 20–24 years. The parental factors emerged across all the six groups interviewed with the sub-theme of parents sharing information late only emerging among male and female groups of ages 10–14 years. The healthcare workers factor only emerged from two FGD sessions with boys ages 15–19 years. All six groups interviewed in both sex and age groups identified teacher factors as barriers to accessing SRH information and services.

### Individual factors

The AY described themselves as being fearful of obtaining SRH treatment due to the personal and social stigma connected with SRH concerns. They believed they were too young for SRH information and worried being assessed based on the SRH information they requested (Q1 & 2).

### Parental factors

Parenting was reported by participants to be a contributor to and cause of numerous barriers to receiving SRH knowledge among the AY. Participants reported that parents perceived AY to be unprepared for SRH information and services, and that parents shared SRH information late (Q3&4).

They were also reported to believe that providing SRH information will likely induce AY to engage in sexual behaviors (Q5-8). According to the AY, some parents think it shameful to openly disclose SRH information with AY (Q9&10).

Participants stated that some AY were orphans with no guardians to provide the necessary SRH information, while others had parents who did not have time to discuss SRH issues with their children, and others may not be enrolled in school at all. These were seen as missed opportunities for the AY’s access to SRH information (Q11-14).

Participants also observed that gender, culture, and religion influenced AY access to SRH information, with fathers rarely providing SRH information to daughters and mothers rarely providing SRH information to sons (Q15-17). Conflict with parents was also shown to limit opportunities for SRH dialogue with the AY (Q18).

Participants reported that some parents assumed that the AY gathered SRH information from other sources, such as the school environment, and thus did not need to discuss it with the parents (Q19), while others believed that this was due to some parents being illiterate and thus deferring SRH engagement to other socializing agents in society, and that the AY was well informed about SRH (Q20).

### Health care worker and health institution factors

The age and gender of health care workers were identified as contributing to or causing barriers to receiving SRH information among the AY. They found it challenging to discuss SRH issues with elderly healthcare workers, particularly men (Q21).

Access fees was also reported as a barrier. Participants sited public facilities to offer free SRH services, whereas the same services were available at a fee in private facilities. Despite mentioning free services in public hospitals, they mentioned having to pay informal fees during a hospital visit, such as fees for consultation, drugs, and laboratory tests. Those without money were unlikely to have access to the services needed (Q 22&23).

While some facilities provided services every week, participants claimed that several health facilities did not provide services on weekends. Some services were only available during specific hours of the day, which was inconvenient for AY and made access difficult (Q 24–26).

Furthermore, the excessive wait times and frequent absence of healthcare staff rendered these services unappealing to the AY (Q27).

Health facilities were described as few and located far from users. Participants noted that some health facilities were accessible; however, one required transport fees (Q29-30).

### Teacher factors

The participants noted that their teachers provided them with limited SRH information. They stated that this was maybe due to the fact that they were students, therefore teachers offered them limited knowledge (Q31&32), or due to the teachers fearing to upset the parents or for fear of teachers losing their jobs(Q33&34). The participant also reported that the time allocated to discussing SRH in schools was limited (Q35).

### Contextual factors

These were mostly identified as literacy challenges in the community, cultural and religious barriers, and poverty. These subthemes were articulated across all six groups of participants of both sexes.

Participants reported a low or complete lack of SRH education and understanding in the community, all of which are associated with low literacy. This was especially concerning when people were illiterate and living in marginal rural areas (Q36&37).

In terms of culture, participants stated that community members followed the teachings of traditions and religion, and that SRH knowledge was ideally shared only after marriage. As such the AY was not expected to have access to this information (Q38&39).

Poor AY were regarded to have restricted access to SRH information. The AY from better-off community members were perceived to have more opportunities to obtain this information from peers and parents than those from poorer homes (Q40-44).

### Factors facilitating access of SRH information and services among AY

The following six factors were identified by the participants as factors that facilitate access to AYSRH in the coastal counties. They included AYSRH sessions in school, supportive parenting, peer support, a supportive health system, using social media to promote AYSRH, and incorporating gender inclusivity in AYSRH programming.

### AYSRH sessions in schools

Participants described that schools provided opportunities for the AY to get information on AYSRH. These were mainly delivered by visiting organizations or health workers (Q45-47).

### Supportive parenting and culture

SRH education within the coastal region is reported to be culturally gendered, whereby boys were to be taught by men/fathers and girls were to learn from women/mothers. As such after the child reaching puberty the culture and religion allows the parents to educate their children on such matters (Q48).

Educated families and those with a positive child-parent interaction were more likely to provide SRH information for AY. AY had a difficult time approaching strict parents for SRH knowledge and help. However, when parents were approachable, AY reported their SRH challenges and received assistance in developing practical answers (Q49).

### Peer support

The AY found it easy to communicate amongst themselves, especially given the difficulties highlighted in the parent-AY relationship. Those who were close, for example, friends, were described as being able to share their personal problems (Q51-53).

### Supportive health institutions

The health facilities were stated to be places where SRH information could be accessed. At the health facilities, HCW would start services by giving health education which sometimes covered topics around SRH (Q54). The HCWs were also reported as ready to assist the AY who came for care; however, sometimes they criticized the AY, which made them uncomfortable seeking SRH services (Q55). Participants also reported some health facilities having started creating AY-friendly services that were separated from the main services offered at the facility and expressed the need for such services to be advertised far and wide so that AY can benefit.

### Digital technology for AYSRH

Media technologies were highlighted as viable venues for reaching and interacting with AY on SRH issues. The digital platforms were seen to bridge the gap of illiteracy in the community and at home, as well as limited information offered in schools or health facilities (Q56).

### Gender inclusivity

Participants mentioned that the perception that girls needed more SRH support than boys also seemed to influence who and how they received SRH information. As such, the participants felt that a lot of attention was being directed to the girls, yet adolescent boys also needed SRH information but were left out of SRH efforts (Q57).

## Discussion

This study contributes to the understanding of the challenges and opportunities for accessing SRH information and services among adolescents and youth (AY) in a low-resource setting. The results confirm the themes from previous studies where parental, teacher, and healthcare workers and health institution factors were identified as barriers to accessing AYSRH services. Contextual factors such as poverty, culture, religion and literacy in the community were also identified as barriers. AYSRH sessions in schools, supportive parenting and culture, peer support, supportive health institution, digital technology, and gender inclusivity in AYSRH programming were considered important for facilitating access to AYSRH information and services in the coastal region.

Participants in our study reported that the age and gender of the health worker is a barrier to AY accessing SRH information and services. They stated that the AY did not feel free to seek SRH information when served by an older health care worker and particular the men.

Educator/ teachers factors were also mentioned. Whereby the participants in this study felt that the educators/teachers share inadequate SRH information. This were mainly perceived as a result of them judging that the information may be too much for the AY or for fear of receiving blame from the parents and the community as well as for fear of losing their jobs. These perceptions could be explained by the current socio-political context in Kenya on matters comprehensive sexuality education (CSE), where, despite the health and education sector emphasizing the right to access AYSRH services and information, there are restrictions on the content and services that can be provided to the school going AY below the age of 18 years, as well as the capacity of the teachers to provide these services [18]. Similar findings were also reported in a systematic review conducted in low resource setting where parents, teachers and health care workers despite being trusted by the AY to offer SRH information gaps existed in the getting these information to them through the education institutions or pathways [19, 20].

Other factors mentioned in the study as barriers to receiving SRH information and services were AY individual factors, cultural and religious beliefs, community literacy, and poverty. Systematic reviews of adolescent pregnancies in low-resource settings point to the role of harmful religious and cultural practices, such as early marriages [12], low socioeconomic status, low literacy levels, lack of agency, and empowerment among adolescents in the resulting poor AYSRH [20, 21].

In addition to these barriers, this study also sought to identify opportunities for improving access to AYSRH information and services. Several factors facilitating access to AYSRH have been identified. They included provision of AYSRH sessions in schools, positive health care workers and institution practices, peer support, supportive parenting and culture, digital technologies as alternative pathways for providing information, and gender inclusivity. All of these factors fall within what is termed as youth-friendly sexual and reproductive health services [22].

In this study, supportive parenting was a significant factor of access to AYSRH. The study’s findings highlight the importance of parents being friendly to the AY in order to facilitate SRH discussions, and they were viewed as a protective factor in preventing adolescents from engaging in sexual risk behavior. These findings are consistent with findings from a systematic review on parent-adolescent communication in Ethiopia, where having a good relationship between the parent and the AY acted as a protective factor and reduced the AY’s engagement in risky behavior [12, 13]. They are also in line with the World Health Organization’s recommendations on the critical role of parents in adolescents’ health and development [3]. In this study, however, culture and religion was seen to have an influence on parenting where only fathers/men within a family unit being reported to have the approval to provide information and guidance to a boy child around the issues of AYSRH, while only women/mothers can teach girls.

Outside of the family setting, peer support was identified as a facilitator of AYSRH information and services in this study. However, studies have found that peer education has an impact on SRH knowledge and attitude, but not on behavioral outcomes [14].

AYSRH sessions in school was another factor identified for improving the AYSRH on the Kenyan coast. Evidence shows that teachers and the school environment are powerful authorities and institutions that can profoundly shape AYSRH in a positive manner [23] and act as structures through which age-appropriate and culturally relevant teaching of sexuality and relationships can be provided [24].

Gender on AYSRH programming was not sought after in this study; however, it did come out in one of the discussions where the men felt the AYSRH programs focused more on women than men, and thus recommended the need to identify and modify intervention components and processes to ensure that both boys and girls are involved. This finding is consistent a done in Kenya that showed that gender inclusivity is a strategy for improving SRH outcomes among the AY in this context.

## Limitations

This study has several limitations. First, the focus group discussion method revealed perceptions rather than actual behaviors. Another limitation is the overrepresentation of women over men in our sample, where more women showed up in the FGDs for those aged 20–24 years and those with CHVs, which may have missed some of the gendered issues on matters AYSRH among these groups. Another limitation is the use of convenience sampling, which may have reduced the representativeness of the findings. Nonetheless, we attempted to include all scenarios of both AY in education and those not in education.

## Conclusions

The study demonstrates that adolescents and youth’s access to AYSRH services and information is heavily influenced by a variety of individual, social, cultural, and economic issues, necessitating the involvement of various stakeholders and interventions. Within the coastal counties, intercounty differences were observed in the findings, with some counties expressing more of some barriers than others, and as such it would be important to take a closer look into the individual counties to identify the specific mechanisms that cause the differences in perception and experiences in future studies. Overall, parents, teachers and education institutions, peer support, gender inclusion, digital technology, supportive health care workers, and health institutions factors are critical in facilitating access to AYSRH information and services.

## Data Availability

All data that support the findings of this study are in the custody of Aga Khan University, Centre of Excellence in Women and Child Health, and are available on request to be made to Evaline Lang’at.

## List of abbreviations

AKU: Aga Khan University
AY: Adolescent and Youth
AYSRH: Adolescent and Youth Sexual and Reproductive Health
AYSRHR: Adolescent and youth sexual and reproductive health and rights
CHV: Community Health Volunteers
FGD: Focus Group Discussion
HCW: Health care workers
JKP: Jumuiya ya kaunti za pwani
LAPSSET: Lamu Port South Sudan-Ethiopia Transport
LMIC: Low-and-middle income countries
SGBV: Sexual and gender based violence
SRH: Sexual and reproductive health
SSA: Sub- Saharan Africa
STI: Sexually transmitted infection
TA: Technical assistance
WHO: World Health Organization

## References

[1] UN World population. prospects: the 2015 revision, key findings and advance tables. (ESA/P/WP 241). 2015.

[2] Habti HE. Why Africa’s youth hold the key to its development potential: World Economic Forum; 2022 [Available from: https://www.weforum.org/agenda/2022/09/why-africa-youth-key-development-potential/

[3] Liang M, Simelane S, Fillo GF, Chalasani S, Weny K, Canelos PS, Jenkins L, Moller AB, Chandra-Mouli V, Say L, Michielsen K (2019). The state of adolescent sexual and reproductive health. J Adolesc Health.

[4] Manguro G, Temmerman M (2022). A critical review of adolescent sexual and reproductive health and rights in Kenya. Med.

[5] Cowden RG, Tucker LA, Govender K. Conceptual pathways to HIV risk in Eastern and Southern Africa: an integrative perspective on the development of young people in contexts of social-structural vulnerability. Preventing HIV Among Young People in Southern and Eastern Africa. 2020:31–47.

[6] Kenya National Bureau of Statistics. 2019 Kenya population and housing census In: Kenya National Bureau of Statistics, editor. Nairobi, Kenya. 2019.

[7] Santhya KG, Jejeebhoy SJ (2015). Sexual and reproductive health and rights of adolescent girls: evidence from low- and middle-income countries. Glob Public Health.

[8] Ministry of Health (2018). Kenya Population-based HIV Impact Assessment (KENPHIA) 2018.

[9] KNBS ICF (2023). Kenya Demographic and Health Survey 2022: key indicator report. Nairobi, Kenya and Rockville.

[10] Jumuiya ya Kaunti Za Pwani. A Kenya coast regional bloc that creates wealth for its community. 2022 [Available from: https://jumuiya.org/coast/

[11] Ssewanyana D, Mwangala PN, van Baar A, Newton CR, Abubakar A (2018). Health Risk Behaviour among adolescents living with HIV in Sub-saharan Africa: a systematic review and Meta-analysis. Biomed Res Int.

[12] Plourde KF, Fischer S, Cunningham J, Brady K, McCarraher DR (2016). Improving the paradigm of approaches to adolescent sexual and reproductive health. Reproductive Health.

[13] Mekie M, Addisu D, Melkie A, Taklual W (2020). Parent-adolescent communication on sexual and reproductive health issues and its associated factors in Ethiopia: a systematic review and meta-analysis. Ital J Pediatr.

[14] Siddiqui M, Kataria I, Watson K, Chandra-Mouli V (2020). A systematic review of the evidence on peer education programmes for promoting the sexual and reproductive health of young people in India. Sex Reprod Health Matters.

[15] Ruane-McAteer E, Gillespie K, Amin A, Aventin Á, Robinson M, Hanratty J (2020). Gender-transformative programming with men and boys to improve sexual and reproductive health and rights: a systematic review of intervention studies. BMJ Global Health.

[16] QSR International Pty Ltd. NVivo qualitative data analysis Software Version 10. 2014.

[17] Braun V, Clarke V. Thematic analysis: American Psychological Association; 2012.

[18] Obare F, Birungi H (2013). Policy scripts and students’ realities regarding sexuality education in secondary schools in Kenya. Sex Educ.

[19] Munakampe MN, Zulu JM, Michelo C (2018). Contraception and abortion knowledge, attitudes and practices among adolescents from low and middle-income countries: a systematic review. BMC Health Serv Res.

[20] Denno DM, Hoopes AJ, Chandra-Mouli V (2015). Effective strategies to provide adolescent sexual and reproductive health services and to increase demand and community support. J Adolesc Health.

[21] Yakubu I, Salisu WJ (2018). Determinants of adolescent pregnancy in sub-saharan Africa: a systematic review. Reproductive Health.

[22] Ninsiima LR, Chiumia IK, Ndejjo R (2021). Factors influencing access to and utilisation of youth-friendly sexual and reproductive health services in sub-saharan Africa: a systematic review. Reproductive Health.

[23] Chavula MP, Zulu JM, Hurtig AK (2022). Factors influencing the integration of comprehensive sexuality education into educational systems in low- and middle-income countries: a systematic review. Reprod Health.

[24] Browes NC (2015). Comprehensive sexuality education, culture and gender: the effect of the cultural setting on a sexuality education programme in Ethiopia. Sex Educ.

